# Long-Term Survival of Cellulose Sulphate-Encapsulated Cells and Metronomic Ifosfamide Control Tumour Growth in Pancreatic Cancer Models—A Prelude to Treating Solid Tumours Effectively in Pets and Humans

**DOI:** 10.3390/life13122357

**Published:** 2023-12-18

**Authors:** Brian Salmons, Walter H. Gunzburg

**Affiliations:** 1 Austrianova Singapore Pte Ltd., 2 International Business Park, The Strategy @ IBP #09-04, Singapore 609930, Singapore; salmons@sgaustria.com; 2 Institute of Virology, Department of Pathobiology, University of Veterinary Medicine, 1210 Vienna, Austria

**Keywords:** cell therapy, encapsulated cells, GDEPT, metronomic, tumour therapy, pancreatic cancer, solid tumours, long-term expression, targeted therapy, stable cell lines

## Abstract

Background: The use of encapsulated cells for the in vivo delivery of biotherapeutics is a promising new technology to potentiate the effectiveness of cell-based therapies for veterinary and human application. One use of the technology is to locally activate chemotherapeutics to their short-lived highly active forms. We have previously shown that a stable clone of HEK293 cells overexpressing a cytochrome P450 enzyme that has been encapsulated in immunoprotective cellulose sulphate beads can be implanted near solid tumours in order to activate oxazaphosphorines such as ifosfamide and cyclophosphamide to the tumour-killing metabolite phosphoramide mustard. The efficacy of this approach has been shown in animal models as well as in human and canine clinical trials. In these previous studies, the oxazaphosphorine was only given twice. An analysis of the Kaplan–Meier plots of the results of the clinical trials suggest that repeated dosing might result in a significant clinical benefit. Aims: In this study, we aimed to (i) demonstrate the stable long-term expression of cytochrome P450 from a characterized, transfected cell clone, as well as (ii) demonstrate that one implanted dose of these encapsulated cytochrome P450-expressing cells is capable of activating multiple doses of ifosfamide in animal models. Methodology: We initially used cell and molecular methods to show cell line stability over multiple passages, as well as chemical and biological function in vitro. This was followed by a demonstration that encapsulated HEK293 cells are capable of activating multiple doses of ifosfamide in a mouse model of pancreatic cancer without being killed by the chemotherapeutic. Conclusion: A single injection of encapsulated HEK293 cells followed by multiple rounds of ifosfamide administration results in repeated anti-tumour activity and halts tumour growth but, in the absence of a functioning immune system, does not cause tumour regression.

## 1. Introduction

The use of suicide gene prodrug combinations, also known as gene-directed enzyme prodrug therapy (GDEPT), is a common strategy for the treatment of solid tumours [1,2]. We have been focusing on a modified form of GDEPT where encapsulated cells expressing the enzyme encoded by the cytochrome P450 2B1 gene are placed in the vicinity of tumours in order to locally activate prodrugs of the oxazaphosphorine family such as ifosfamide and cyclophosphamide [3]. Local activation is required for maximum anti-tumour effects since the CYP 2B1-activated forms of the oxazaphosphorines (4-hydroxy-amides) are short-lived and need to pass through the cell membrane of tumour cells before they spontaneously decay to the anti-tumour active phosphoramide mustard and the tissue toxic form, acrolein [4]. Phosphoramide mustard causes DNA alkylation and irreversible cross-linkages between and within strands at the guanine N-7 position [5]. DNA cross-linking is not toxic per se, but when cells attempt to divide, they die [6]. Although DNA alkylation and cross-linking occur in many cells, its cytotoxic effects are thus limited to rapidly dividing cells such as tumour cells. The intra- and interstrand DNA linkages are, however, also cytotoxic since besides blocking DNA replication, they also block essential processes such as DNA transcription [7,8]. The mechanism of cell death has been thought to be via apoptosis, although death primarily via necrosis, rather than apoptosis, has been shown in cell culture [9]. 

The encapsulation of contact-inhibited cells at high cell density not only confines them to the site at which they are implanted and protects them from clearance by the host immune system, but it also prevents their replication, so that they themselves are not killed by the activated prodrug [4,10,11].

Local encapsulated cell-mediated activation has been shown to give better anti-tumour effects (tumour shrinkage, enhanced median survival) even when low doses of oxazaphosphorines, such as ifosfamide, are used, thereby mitigating the usual side effects that are observed with standard doses in two human clinical trials for pancreatic cancer [12]. These data are supported by another clinical trial using encapsulated cell-mediated local activation of cyclophosphamide in dogs with spontaneously occurring mammary cancer [13].

The previously reported clinical trials of this encapsulated cell therapy for the treatment of pancreatic cancer were performed using one administration of encapsulated CYP 2B1-expressing cells, and subsequently, two rounds of systemic ifosfamide treatment [12]. However, since the cells in the capsule are non-dividing but metabolically active, they should be able to continue to convert multiple doses of ifosfamide. In this study, we wanted to determine whether (i) encapsulated cells in general can survive and remain viable for long periods after implantation into mice, and (ii) whether multiple rounds of ifosfamide can be delivered and activated by the encapsulated cells without the encapsulated cells eventually being killed.

## 2. Materials and Methods

### 2.1. Parental HEK293 Cell Lines

The parental cell line HEK 293 (transformed primary embryonal kidney, human) is certified by the American Type Culture Collection (ATCC). ATCC forwarded the cell line to the European Collection of Animal Cell Cultures (ECACC), from where it was provided to Q-One (UK), now Invitrogen, for cell banking and adventitious agent testing. The cells were stored at Q-One under cGMP conditions.

### 2.2. Transfection with Expression Vector and Selection of Clones

For preparation of the CBT-4B10 cell line, one “Batch 3/Passage 7” vial of HEK293 cells was thawed and expanded for 10 days in Dulbecco’s Modified Eagle’s Medium (DMEM) + 10% Foetal Bovine Serum (FBS). Parental cells were then seeded for transfection into 6-well plates. One day later, cells were transfected with the expression plasmid pCMV.CYP2B1 using the calcium phosphate method. The plasmid pCMV.CYP2B1 contains the rat CYP2B1 gene (CYP2B1) under transcriptional control of the human cytomegalovirus promoter (CMV) with the bovine growth hormone polyadenylation signal (BGH polyA). Additionally, the plasmid contains an ampicillin resistance gene (amp), as well as a neomycin resistance gene (Neo) that can be selected for after transfection into mammalian cells using geneticin (G418). Transfection was carried out according to the standard protocol using a transfection kit (Cell Phect, GE Healthcare, Chicago, IL, USA). The following day, 100 cells were seeded in 96-well plates, and subsequently, transfected cells were selected in DMEM + 10% FBS containing 400 Mg geneticin (G418, Invitrogen, Lofer, Austria). After one month of selection with regular medium changes, individual clones were transferred to individual wells of a 6-well plate and expanded further under selection. 

For preparation of the 293-luc cell lines, HEK293 cells were seeded for transfection into 6-well plates and grown in DMEM with Glutamax (Invitrogen) supplemented with 10% FBS (Invitrogen). Transfections were performed using Lipofectamine 2000 (Invitrogen) as recommended by the supplier. The plasmid pCMV.hyg.luc contains the luciferase gene (luc) under the transcriptional control of the human cytomegalovirus promoter (CMV), as well as the bacterial selection marker hygromycin. Transfected cells were selected in medium containing 0.2 mg/mL hygromycin (Invitrogen) until mock-transfected cells had died. Stably transfected cells were maintained as populations in the presence of 0.2 mg/mL hygromycin. 

### 2.3. IVIS Imaging

Six Cell-in-a-Box capsules containing ~6 × 10^3^ 293-luc cells per capsule were implanted subcutaneously (sc) near the left scapula, and another 6 intraperitoneally (ip) into each of 15 female nude mice (Hsd: Athymic Nude (Foxn1nu)) using glass capillaries. Prior to imaging, 240 Mg of luciferin (Xenogen) per Kg of body weight was injected ip into anaesthetised mice (isoflurane). In vivo images were acquired using an IVIS 50 (Xenogen Corp., Alameda, CA, USA) and analysed using IGORpro software Version 5.1 (WaveMetrics, Inc. Lake Oswego, Oregon, USA.) (unpublished conference abstract).

**Figure 1:**
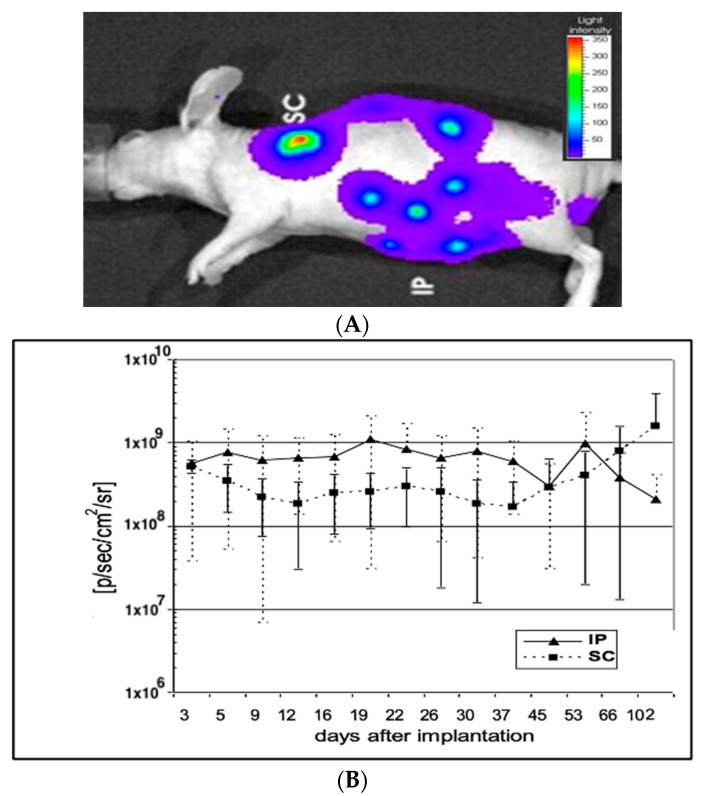
(**A**) Detection of ip and sc implanted capsules containing 293luc cells. Red colours indicate high concentrations of encapsulated cells. 293luc cells encapsulated in six 0.7mm diameter cellulose sulphate capsules were injected either subcutaneously (sc) or intraperitoneally (ip) into 3 mice. Twenty minutes before detection, luciferin was injected ip and the capsules were visualised under an IVIS 50. (**B**) Luciferase activity of intraperitoneally (ip) and subcutaneously (sc) implanted capsules containing immobilised 293luc cells. The mice shown in Figure 1 were subjected to IVIS 50 measurement every few days and the detected signal strength was plotted. Mice injected with 6 capsules intraperitoneally (ip) showed a remarkably constant signal (p) over 100 days post implantation, whilst mice injected with 6 capsules subcutaneously (sc) showed a slightly lower, but again, constant signal (¢) over the first month, which then began to increase.

The first IVIS luciferase measurement was performed one day after implantation. Subsequent measurements were performed 8 days, 15 days, 22 days, 29 ± 2 days, 41 ± 2 days, 55 ± 2 days, 69 ± 2 days, 83 ± 2 days and 113 ± 3 days after implantation. The detected signal strength was either expressed as a colour scale (Figure 1A) or plotted out numerically (Figure 1B).

### 2.4. Resorufin CYP2B1 Enzymatic Expression Assay

Individual clones were tested using a resorufin assay in which the amount of CYP2B1 enzymatic activity was evaluated using a surrogate substrate, 7-pentoxyresorufin, which is converted to resorufin via CYP2B1 activity as previously described [14].

### 2.5. Western Blotting of CYP2B1 Protein

**Figure 2:**
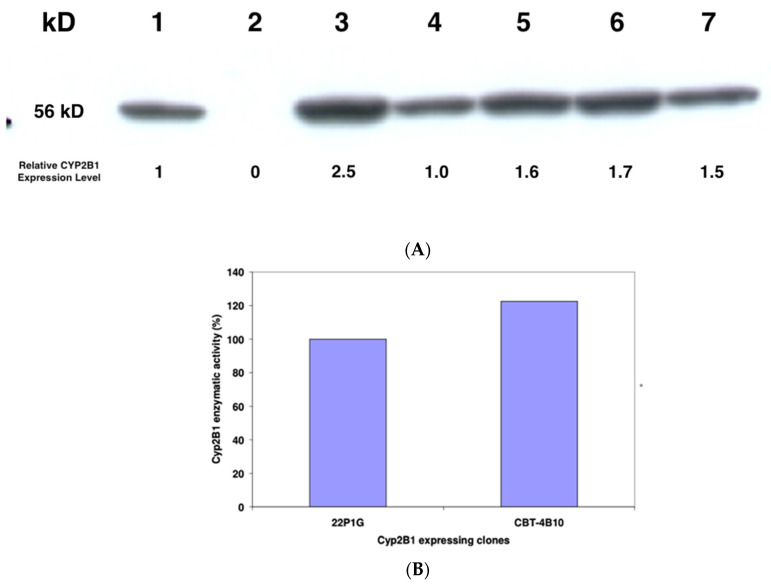
(**A**) Semi-quantitative Western blot analysis of steady-state CYP2B1 protein levels in 22P1G, and the five candidate cell clones. A total of 10% crude protein lysate from 1 × 10^6^ 22P1G (lane 1), 293 (lane 2), CBT-3D7 (lane 3), CBT-4H9 (lane 4), CBT-1B6 (lane 5), CBT-4F5 (lane 6) and CBT-4B10 (lane 7) cells was applied to the SDS-PAGE, and after electrophoresis and transfer of the proteins to a membrane, the blot was probed with an antibody that is specific for CYP2B1 (Becton Dickinson). (**B**) CYP2B1 enzymatic activity. Resorufin assays to determine the enzymatic activity of CYP2B1 in 22P1G or CBT-4B10 cells, respectively. Substrate turn-over was estimated after the addition of 15 mM 7-Pentoxyresorufin.

Semi-quantitative Western blotting was performed essentially as outlined by Lengler and colleagues [14] to show the presence of CYP2B1 protein in the cells. Crude protein lysate from 1 × 10^6^ cells of each clone was prepared, and ten percent applied to an SDS-PAGE gel, along with extract from nontransfected HEK293 cells and from a standard CYP2B1-expressing cell line, 22P1G (Figure 2A). After electrophoresis and electrophoretic transfer to a nylon membrane, the gel was stained with Coomassie blue and a densitometric analysis performed on the stained gel to correct for protein loading. This analysis revealed that the relative amount of protein loaded in lanes 1 to 7 was 1.5, 1.4, 1.9, 1.5, 1.4, 1.3 and 1 respectively. The membrane was probed with a polyclonal goat anti-CYP2B1 antibody diluted 1:5000 (Becton Dickinson) and a densitometric analysis performed on the CYP2B1-specific bands (Figure 2A), to de-termine the relative expression levels of CYP2B1 in the various cell lines compared to the 22P1G cell clone (lane 1, set as 1). The calculated relative CYP2B1 expression levels are also shown in Figure 2A.

### 2.6. XTT Metabolic Activity Assay 

The XTT assay [15] was performed by seeding 5 × 10^3^ cells/well for each cell line in quadruplicate into 96-well plates. The cells were grown in DMEM + 10% FBS with the addition of 0, 0.005, 0.015, 0.05, 0.165 or 0.54 mM ifosfamide for 5 days, and the metabolic activity of cells was then measured (Table 1). The IC_50_ of the clones was evaluated using Excel Fit.

### 2.7. Cell-in-a-Box Encapsulation

Capsules were produced as described previously [11]. In brief, 1 × 10^7^ cells were suspended in 1 mL Phosphate-Buffered Sulphate (PBS) (pH 7) containing 2–5% cellulose sulphate (Austrianova, Singapore) and 5% FCS (Gibco, Waltham, MA USA). The suspension was allowed to drop freely from an adjustable dispersion system by regulating the flow into a precipitation bath containing 3% polydiallyldimethyl ammonium in PBS. Capsule formation occurred within milliseconds, followed by further constitution of an inner, more porous, layer for mechanical support. The capsules were washed twice with normal DMEM medium, and then, added to tissue culture until the capsules were full (around 14 days). Capsules were maintained in serum-free medium prior to application in mice.

### 2.8. Cell Killing via “by-Stander” Effect

On day 1, approximately 5 × 10^3^ Crandell feline kidney (CrFK) cells were seeded in 96-well plates in quadruplicate. On day 2, ten (10) capsules of Cell-in-a-Box encapsulated cells were added and treated with 0, 0.5 and 0.75 mM ifosfamide, respectively, as previously described [16]. After removal of the capsules on day 7, an AlamarBlue assay was performed to determine the metabolic activity of the recipient cells. 

### 2.9. Tumour Cell Line

The human pancreatic carcinoma cell line PaCa-44 (ATCC), derived from a typical adenocarcinoma of moderate-to-poor differentiation (G2–3), was used. This cell line has been extensively characterized by us and others [17,18,19]. The cell line carries mutations in codon 12 of the ras oncogene, as well as in exon 5/6 of the p53 tumour suppressor gene, but it has a wild-type RB1 gene. PaCa-44 cells were grown in Roswell Park Memorial Institute (RPMI) cell culture medium supplemented with 10% FCS, penicillin and streptomycin (Gibco). 

### 2.10. Animal Experiments

Proliferating PaCa-44 cells were used to establish tumours in the nude mouse. A total of 1 × 10^6^ cells were injected subcutaneously in the flanks of nude mice (CD-1 nu/nu; Bioservice) in RPMI without supplements [17]. The cells were suspended in 100 μL RPMI, added to a 1 mL standard syringe (Braun, Melsungen, Germany) and injected through a 27 G needle (Microlance 3; Becton Dickinson, Fraga, Spain). Experiments were started with tumours of comparable size (200–500 mm^3^). Capsules of encapsulated cells were delivered through a venous catheter (Vasocan Braunüle 18G) directly into the tumour. Approximately 80–100 capsules were delivered per injection. Animals were treated intraperitoneally every third day for 2 weeks with ifosfamide at 100 mg/kg body weight (Holoxan; Asta Medica, Frankfurt-am-Main, Germany). At the same time, sodium 2-mercaptoethanesulphonate (MESNA; Asta Medica) was administered at the same dosage (100 mg/kg body weight) ip to protect the urinary tract. 

### 2.11. Statistical Analysis

To evaluate the significance of the differences between the means of the replicates, a two-tailed *t*-test was performed. The differences between the means at each time point were calculated, and the null hypothesis that the difference is zero applied. Using software from MedCalc [20] and inputting the difference between the means, the standard deviation and the group size, the probability of obtaining the calculated difference between the means if the null hypothesis was correct was calculated [21,22]. If a *p* value of less than 0.05 (*p* < 0.05) was obtained, the two means were regarded as significantly different, whilst a *p* value of less than 0.0001 (*p* < 0.0001) demonstrated a highly significant difference.

## 3. Results

### 3.1. Mice Implanted with Encapsulated Cells Expressing Luciferase

In order to investigate the viability of encapsulated cells in vivo, cellulose sulphate-encapsulated HEK293 cells containing and constitutively expressing luciferase (293luc) were implanted into mice and imaged using an IVIS imaging system. Initially, the luciferase-expressing 293luc cells were implanted into immunocompetent mice, but autofluorescence from the mouse hair masked the imaging of luciferase expression in vivo, as has been seen by others [23,24]. We thus turned to nude mice, where there is little-to-no background fluorescence. The encapsulated luciferase-expressing cells were implanted at two sites: (i) subcutaneously (sc) and (ii) intraperitoneally (ip). At each site, six capsules were implanted. In vivo bio-imaging of animals after the application of luciferin revealed that the encapsulated HEK293 cells expressing luciferase that were implanted subcutaneously into the left shoulder remained localised. In contrast, intraperitoneally implanted encapsulated HEK293 cells expressing luciferase could be found at different locations in the abdomen, indicating that they are freely moving in the abdominal space (Figure 1A). As expected, control capsules containing HEK293 cells do not fluoresce. A quantitative analysis of luciferase expression from the encapsulated, luciferase-expressing HEK292 cells revealed that regardless of the site of implantation expression levels remained consistently high over at least a 102-day period (Figure 1B). These results indicate that encapsulated HEK cells survive more than 3 months in mice and that the capsule membrane remains structurally intact, promoting the long-term survival of immobilised cells in vivo.

### 3.2. Model for Anti-Tumour Use of Encapsulated Cells

To demonstrate the utility of using encapsulated cells expressing an active molecule over long periods for tumour treatment, the PaCa44 model was used. In this model, a subcutaneous human-derived pancreatic carcinoma is established in the mouse, and then, treated with the activated products of ifosfamide. Ifosfamide, a member of the oxazaphosphamide family, is systemically administered, and then, activated by cytochrome P-540 enzymes, expressed predominantly in the liver, but also present in other tissues, to its activated 4-hydroxy-ifosfamide form, which is able to enter cells but has a very short half-life, spontaneously decaying to acrolein and phosphoramide mustard, which causes DNA-cross-linking and ultimately cell-death [8]. 

Although ifosfamide is indicated for the treatment of pancreatic cancer in the EU and Canada [25,26], it is rarely used, since the doses needed to have a measurable antitumour effect are associated with unacceptable side effects [27,28,29,30,31]. This is because the DNA cross-linking is not restricted to tumour cells, but will affect all cells, leading to death in dividing cells such as those found in the immune system, digestive tract, hair, nails, etc.

The ability to deliver therapeutically active concentrations of cytochrome P450 enzymes close to tumours would open the possibility of the use of lower doses of ifosfamide that would still have a local therapeutic effect, whilst only causing limited side effects [32,33].

Of the cytochrome P450 isoforms known to activate ifosfamide, rat cytochrome P450 2B1 (which is the homolog of the human cytochrome P450 2B1) has been shown to be one of the most promising from a clinical standpoint since it shows little or no conversion to the neurotoxic metabolite, chloroacetaldehyde, compared to the human cytochrome P450 2B1 [34]. For this reason, we engineered a HEK293-based cell line stably expressing cytochrome P450 2B1 at high levels. 

### 3.3. Generation and Characterization of a Stable Cell Line Producing Consistently High Levels of Cytochrome P450 2B1

HEK293 cells were transfected with the expression plasmid pCMV.CYP2B1, which contains an expression cassette, where the cytochrome P450 2B1 isoform is expressed from a cytomegalovirus promoter. Its correct DNA structure has been validated using test digests with several restriction endonucleases and combinations of them. 

After transfection, cells were selected in a medium containing 400 μg/mL geneticin (G418), and individual clones were picked and grown. These individual cell clones underwent two screening procedures. In a first round of screening, expanded cell clones were analysed for CYP2B1 enzymatic activity. The five clones with the highest CYP2B1 expression were further subjected to semi-quantitative Western blot analysis to confirm the presence of CYP2B1 protein (Figure 2A). A 56KD protein that reacted with a CYP2B1 antibody was detected in all tested clones, but not in the parental HEK293 cells. Further, the same protein was detected in the reference cell line 22P1G.

The five CYP2B1-expressing clones were then analysed for their metabolic activity after exposure to different concentrations of ifosfamide (Table 1).

Clone CBT-4B10 was selected since it expressed CYP2B1 protein (Figure 2A) and showed the lowest IC_50_ at 0.0117 mM ifosfamide (Table 1), meaning that it was the most resistant to self-killing induced by activated 5-hydroxy-ifosfamide. The enzymatic activity was found to be around 20% higher than that previously measured in the previously developed standard CYP2B1-expressing cell line 22P1G (Figure 2B).

The analysis of genomic DNA revealed that the clone CBT-4B10 contained a single integration site, with no oncogenes located in the vicinity of the transgene and with a distance of around 15 kb and 63 kb, respectively, to adjacent genes. The analysis of CBT-4B10 subclones provided further evidence that the parental cells are of clonal origin. No changes in the pattern were observed with all three subclones analysed. 

**Figure 3:**
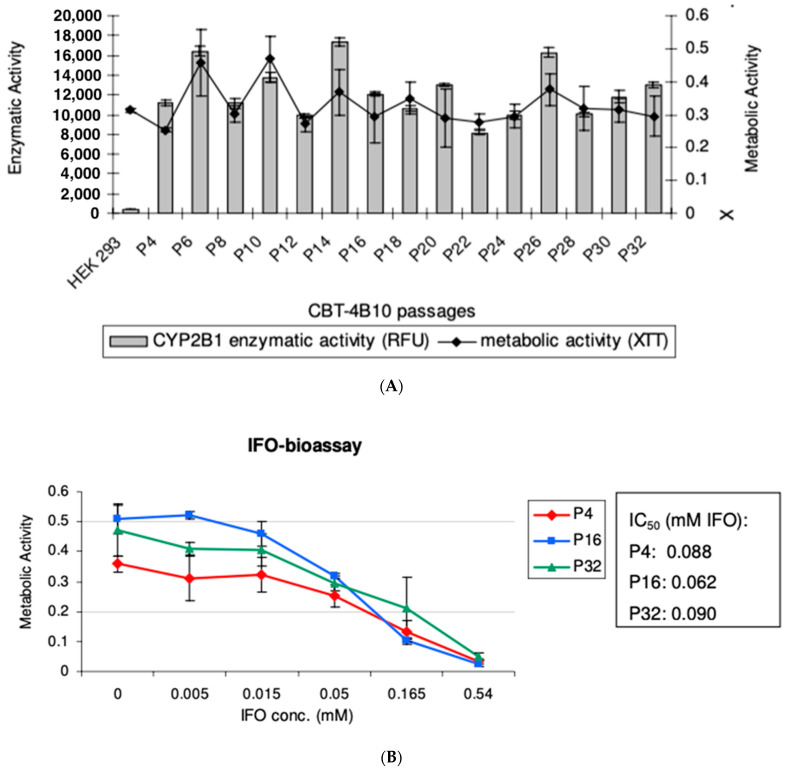
(**A**) Comparison of CYP2B1 enzymatic activity and Metabolic Activity over time for CBT-4B10 cells over Passages 4 to 32. An assay was carried out to evaluate the CYP2B1 enzymatic activity of cells. Additionally, the metabolic activity of these cells was evaluated. The experiment was carried out in quadruplicate and the mean and standard deviation are shown. (**B**) Comparison of CYP2B1 enzymatic activity, with varying ifosfamide concentrations, of CBT-4B10 cells at Passages 4, 16 and 32. An ifosfamide (IFO)-bioassay was carried out to estimate the CYP2B1 bioactivity of different cell passages. Five days after the addition of various concentrations of IFO, the metabolic activity of cells was measured. The IC_50_ of each passage was evaluated using Excel Fit. The experiment was carried out in quadruplicate and the mean and standard deviation are shown.

To evaluate the long-term stability of the CBT-4B10 clone, it was cultured without geneticin selection pressure for 12 weeks up to Passage 32. Cells were compared in terms of metabolic activity and CYP2B1 enzymatic activity at every second passage (P4-P32). Although slight variations could be observed in different passages, no general decrease in metabolic activity and CYP2B1 enzymatic activity, respectively, was observed (Figure 3A).

To confirm these results, early (P4), middle (P16) and late (P32) passages of CBT-4B10 were subjected to an IFO-bioassay, which revealed similar Inhibitory Concentration_50_ (IC_50_) values for the cells of early and late passages (Figure 3B). 

Taken together, these experiments demonstrate that the clone CBT-4B10 showed long-term stability, even without selection pressure, over a long time period. Additionally, the analysis with genomic DNA from cell passages 4, 16 and 32 revealed genetic stability over a 12-week period.

### 3.4. Cytochrome P450 Bystander Activity Mediated by Encapsulated Cells In Vitro

Before embarking on in vivo animal experiments, the CBT-4B10 cells were encapsulated, and then, underwent a bystander assay to simulate the situation in vivo. In this bystander assay, CYP2B1-expressing cells and pancreatic tumour cells were cultured in vitro, sharing the same medium containing ifosfamide. 

**Figure 4:**
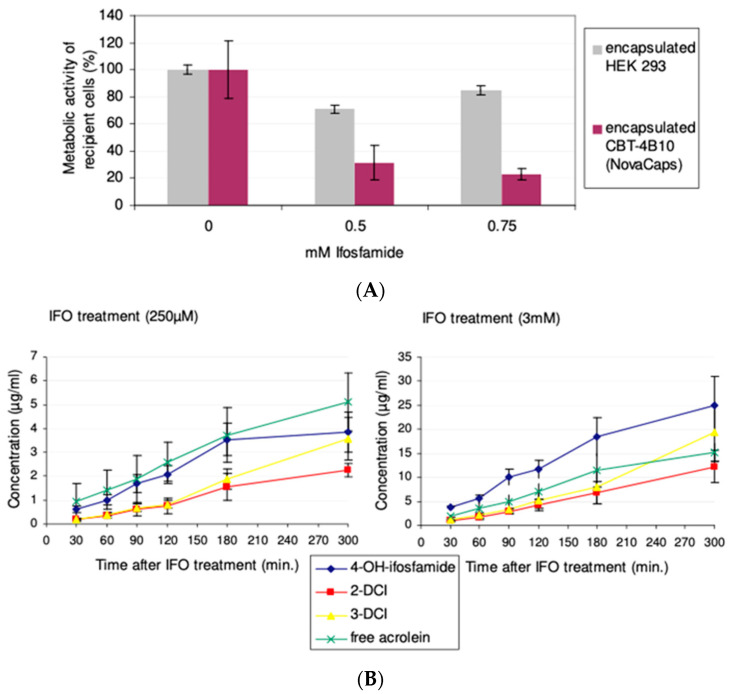
(**A**) Cell-in-a-Box encapsulated CBT-4B10 cells mediated bystander effect at different ifosfamide concentrations: On day 1, approximately 5 × 10^3^ CrFK cells were seeded in 96-well plates in quadruplicate. On day 2, ten (10) capsules—Cell-in-a-Box encapsulated CBT-4B10 cells or encapsulated HEK 293 cells—were added and treated with 0, 0.5 and 0.75 mM ifosfamide, respectively. After removal of the capsules on day 7, an AlamarBlue assay was performed to determine the metabolic activity of the recipient cells. For comparison, the metabolic activity of recipient cells not treated with ifosfamide was set to 100%. The *p* value of the difference in the measured bystander effects were calculated using a two-tailed *t*-test and was found to be significant (*p* = 0.0011) at 0.5 mM ifosfamide, and highly significant (*p* < 0.0001) at 0.75 mM ifosfamide. (**B**) Measurement of metabolites arising from Cell-in-a-Box encapsulated CBT-4B10 cell-mediated ifosfamide activation in vitro; concentrations of ifosfamide metabolites were measured after treatment with 250 µM or 3 mM ifosfamide. The measured metabolites were 4-OH-ifosfamide: 4hydroxyifosfamide (and its tautomeric form aldoifosfamide); DCI: dechloroethylifosfamide; and free acrolein: acrolein without 4-OH-ifosfamide decay. The experiment was carried out in quadruplicate and the mean and standard deviation are shown.

CBT-2B1 cells were encapsulated in cellulose sulphate (Cell-in-a-Box) at a starting cell density of ~400 cells/capsule, and then, expanded in the capsules to a final cell density of ~10,000 cells/capsule. Using a standard assay in which the bystander-killing effect is measured using a reproducible and standardized feline kidney cell (CrFK) target cell line, encapsulated CBT-4B10 cells were co-cultivated with CrFK cells and subjected to treatment with 0.75mM ifosfamide. The CYP2B1 enzyme expressed from the CBT-4B10 cells will convert the ifosfamide to the 5-hydroxy form, which will leave the capsules and enter the target CrFK cells. Here, it will decay to phosphoramide mustard and acrolein, cross-linking nascent DNA strands and moving the CrFK cells into the apoptotic pathway when they try to divide. Encapsulated parental HEK 293 cells served as a negative control. The metabolic activity of feline kidney (CrFK) cells co-cultivated with encapsulated CBT-4B10 was diminished up to 23% when treated with 0.75 mM ifosfamide, whereas the negative control remained nearly stable (Figure 4A).

Encapsulated CBT-4B10 cells were tested for their ability to activate ifosfamide in a time-dependant manner by determining the amount of metabolites over time. After the cultivation of 1000 Cell-in-a-Box encapsulated CBT-4B10 cells in roller bottles for three weeks, 250 µM and 3 mM ifosfamide, respectively, were added. CYP2B1-activated metabolites in the medium were measured via HPLC/VU (4-hydroxyifosfamide and its tautomeric form aldoifosfamide), HPLC/mass spectrometry (2- and 3dechloroethylifosfamide) or via a fluorescence method (acrolein). As a negative control, the parental HEK 293 cell line, not expressing CYP2B1, was also encapsulated, cultivated and treated with 3 mM ifosfamide. No appreciable amounts of acrolein, 2-DCI and 3-DCI could be measured from encapsulated control parental HEK293 cells after 2 h. Only negligible amounts of 4-hydroxyifosfamide (0.016 ± 0.005 µg/mL after 2 h) could be measured in the control HEK293 medium.

In contrast, linearly increasing amounts of all four activated metabolites could be measured in the medium of encapsulated CBT-4B10 cells irrespective of whether the capsules and cells were treated with 250 μM ifosfamide or 3 mM ifosfamide (Figure 4B). However, even though there was a 12-fold difference in the concentration of ifosfamide substrate applied, there was a maximum 5.6-fold increase in the production of 4-hyroxyfosfamide and aldoifosfamide, a maximum 5.7-fold increase in 2-dechloroethylifosfamide, a maximum 4.7-fold increase in 3-dechloroethylifosfamide and a 2.5-fold increase in free acrolein (Table 2), suggesting that the CYP2B1 enzyme produced from the encapsulated cells was saturated by excess substrate at a concentration of 3 mM ifosfamide.

### 3.5. Cytochrome P450 Bystander Activity Mediated by Encapsulated Cells In Vivo

**Figure 5:**
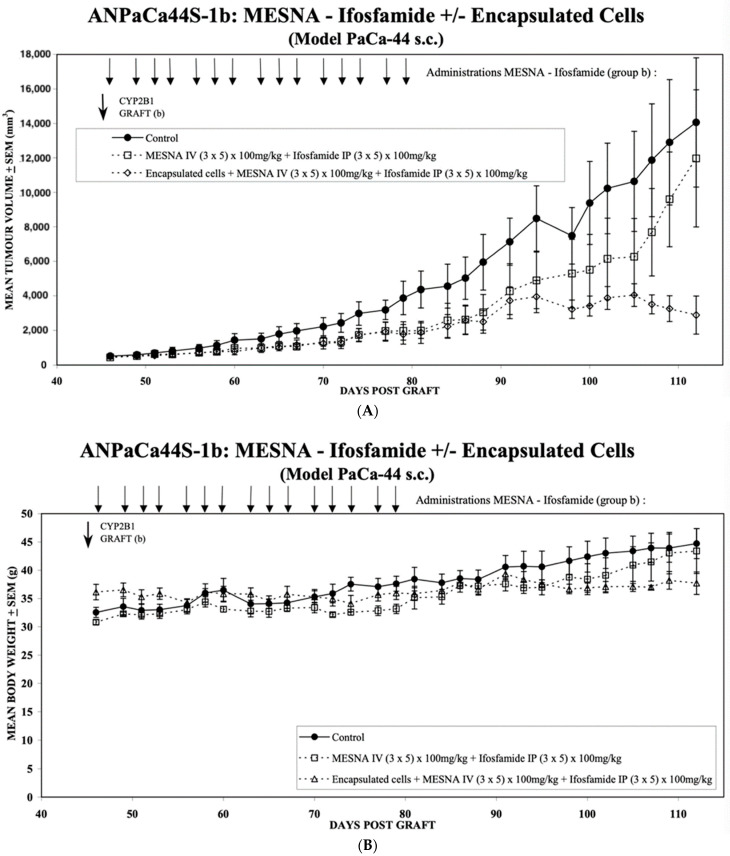
(**A**) Mean tumour volume development over time for the PaCa-44 tumour model with and without local treatment with Cell-in-a-Box-activated ifosfamide. Group 1 was left without treatment (⚫) as the control group. Group 2 was treated with 100 mg/kg ifosfamide intraperitoneally and 100 mg/kg MESNA as a uroprotectant intravenously on days 46, 48, 49, 50, 52, 53, 54, 56, 57, 58, 60, 61, 62, 64 and 65 (☐). Group 3 received capsules of CYP2B1-expressing encapsulated cells (clone 4B10) implanted intratumorally on day 45 (CYP2B1 GRAFT) (b), followed by treatment with 100 mg/kg ifosfamide intraperitoneally and 100 mg/kg MESNA as a uroprotectant intravenously on days 46, 48, 49, 50, 52, 53, 54, 56, 57, 58, 60, 61, 62, 64 and 65 (⟐). Each group consisted of nine mice, and the mean volume and standard deviation are shown. The *p* values of the difference in the means of Group 2 (☐) and Group 3 (⟐) were calculated using a two-tailed *t*-test and were *p* = 0.152 on day 94, *p* = 0.0125 on day 98, *p* = 0.009 on day 100, *p* = 0.0126 on day 102, *p* < 0.0089 on day 105, *p* < 0.0002 on day 107, *p* < 0.0001 on day 109 and *p* < 0.0001 on day 112. (**B**) Mean body weight development over time for the PaCa-44 tumour model with and without local treatment with Cell-in-a-Box-activated ifosfamide. Group 1 was not treated (⚫) and is thus the control group. Group 2 was treated with 100 mg/kg ifosfamide intraperitoneally and 100 mg/kg MESNA as a uroprotectant intravenously on days 46, 48, 49, 50, 52, 53, 54, 56, 57, 58, 60, 61, 62, 64 and 65 (☐). Group 3 received capsules of CYP2B1-expressing encapsulated cells (clone 4B10) implanted intratumorally on day 45, followed by treatment with 100mg/kg ifosfamide intraperitoneally and 100 mg/kg MESNA as a uroprotectant on days 46, 48, 49, 50, 52, 53, 54, 56, 57, 58, 60, 61, 62, 64 and 65 Figure 5A (△). Each group consisted of nine mice, and the mean body weight and standard deviation are shown. The *p* values of the difference in the means of Group 2 (☐) and Group 3 (△) were calculated using a two-tailed *t*-test and were *p* = 0.1485 on day 102, *p* = 0.0103 on day 105, *p* = 0.0074 on day 107, *p* = 0.0037 on day 109 and *p* < 0.0001 on day 112.

Having demonstrated the stable long-term expression of cytochrome P450 2B1 from the HEK293 4B10 clone in vitro, the same encapsulated cells were used to determine their anti-tumour activity in vivo. The flanks of 10 immuno-deficient mice were subcutaneously inoculated with 1 × 10^6^ Paca44 cells, and the tumours allowed to develop for 45 days until they had a mean diameter of 500 mm. At this point, the mice had a mean body weight of 32 g and the mice were randomly allocated to one of three groups. Group 1 was left without treatment (Figure 5A ⚫) as the control group. Group 2 was treated with 100 mg/kg ifosfamide intraperitoneally and 100 mg/kg MESNA as a uroprotectant intravenously (Figure 5A ☐). Group 3 received capsules of CYP2B1-expressing encapsulated cells (clone 4B10) implanted subcutaneously on day 45, followed by treatment with 100 mg/kg ifosfamide intraperitoneally and 100 mg/kg MESNA as a uroprotectant intravenously (Figure 5A ⟐).

The comparison of Group 2 (☐) with Group 1 (⚫) reveals the effect of the mouse endogenous conversion of the applied ifosfamide on tumour growth. There is a clear slowing down of the tumour growth; however, the long-term data (around day 100–110) show that the growth is only inhibited and not abrogated. In contrast, the comparison between Groups 2 (☐) and 3 (⟐) show the added benefit of long-term expression of exogenous Cyp2B1 expressed continuously from the encapsulated 4B10 cells. Here, tumour growth is slowed analogously to the effect of the endogenous conversion (☐) until around day 90, when the conversion by the encapsulated CBT-4B10 cells (⟐) causes tumour growth abrogation and a reduction in tumour size. 

A two-tailed *t*-test was applied to evaluate the statistical significance of the differences in tumour size between the effects of the endogenous conversion of ifosfamide (Group 2 (☐)) and the added benefit of the long-term expression of exogenous Cyp2B1 expressed continuously from the encapsulated 4B10 cells (Group 3 (⟐)). From day 98, the difference between the two groups, one with only the endogenous conversion of ifosfamide (Group 2 (☐)) and the second with the added benefit of exogenous ifosfamide conversion as a result of the implanted encapsulated 4B10 cells (Group 3 (⟐)), was found to be significant (*p* < 0.05).

These results are mirrored by an evaluation of the mean body weight of the mice (Figure 5B). There is a continuous increase in body weight in the control group (⚫), reflecting the increasing size of the tumour. This is paralleled by the mean body weight increase of the endogenous conversion group (☐), initially at a slightly lower weight, but then, as the tumour volume of the endogenous conversion group (☐) more closely matches that of the control group (⚫), around day 100, the two mean body weights are virtually the same. In contrast, the mean body weight of the encapsulated CBT-4B10 cell group (△) starts to decline as the tumour mass shrinks from day 90 onwards. A similar statistical analysis to that applied to the differences between Groups 2 (☐) and 3 (⟐) in Figure 5A was used to test the significance of the differences in mean body weight in Figure 5B. From day 105, the difference between the two groups, one with only the endogenous conversion of ifosfamide (Group 2 (☐)) and the second with the added benefit of exogenous ifosfamide conversion as a result of the implanted encapsulated 4B10 cells (Group 3 (△)), was found to be significant (*p* < 0.05).

## 4. Conclusions

We have shown that cells encapsulated in cellulose sulphate can survive for at least 3 months after implantation into mice and that survival, determined using the surrogate of luciferase marker gene expression, does not appear to depend on the site of implantation (subcutaneous vs. intraperitoneal cavity) (Figure 1A,B). This confirms previous data, such as those obtained from a study where encapsulated rat cells were implanted into the intraperitoneal space in immunocompetent mice [35]. Another previous study conducted in immunocompetent Sprague Dawley rats receiving cellulose sulphate-encapsulated porcine islets and made diabetic via an intraperitoneal injection of streptozotocin were shown to become normoglycemic for 4 months after treatment. After 4 months, encapsulated cells retrieved from the rats were shown to be intact, and immunohistochemical examination revealed that the cells were still viable and producing insulin, demonstrating that even xenogeneic cells survive well when encapsulated and implanted into immunocompetent animals [13,36,37].

A clinical trial of encapsulated cells used to activate cyclophosphamide in dogs with spontaneously occurring mammary cancer resulted in the shrinkage of inoperable tumours so that they became operable. This allowed for the retrieval of the capsules, and viable cells could be detected within the capsule after two rounds of standard-dose cyclophosphamide [13]. Since the cells are still viable, this suggests that multiple rounds of oxazaphosphorines could be given, potentially having enhanced anti-tumour effects. 

In order to develop a system to test this, a new, clonal cytochrome P450-expressing cell line (CBT-4B10) was developed based on HEK293 cells. This cell line was developed to have one integration site of the cytochrome P450 2B1 gene, to produce high levels of CYP2b1 enzyme (Figure 2A) and to show low self-killing activity when exposed to ifosfamide (Table 1). Since these cells should be tested with multiple rounds of ifosfamide, the maintenance of good and consistent ifosfamide expression, along with good cell survival, as measured using metabolic activity, was confirmed over 28 passages (Figure 3A). Since the cells were passaged every 3 to 4 days, this equates to around 100 days. Over this time, there was no loss of ifosfamide expression. Further, the self-killing of the CBT-4B10 cell lines was investigated at the beginning, middle and end of the passage period (Figure 3B). The cell lines showed no significant change in their sensitivity to ifosfamide, as measured using IC_50_ over the tested passages.

The CBT-4B10 cell line showed good killing of co-cultivated CrFK cells in vitro, and the effect was ifosfamide concentration-dependent, with both increased killing (Figure 4A), as well as higher production of measured metabolites (Figure 4B), at higher ifosfamide concentration. The CBT-4B10 cell line was thus deemed to be highly stable and suitable for long-term testing in vivo using multiple rounds of ifosfamide.

To mimic the effect of multiple rounds of ifosfamide in mice, a Paca44 xenograft preformed model was used. PaCa-44 cells were chosen since they give more aggressive and invasive tumours and are an established model for pancreatic carcinoma [17]. A total of 15 doses of ifosfamide and MESNA over 20 days were given in the presence or absence of implanted CBT-4B10 cell-containing capsules. While there was a clear effect arising from mouse endogenous liver-mediated activation of the ifosfamide in slowing tumour growth (Figure 5A, (☐) compared with (⚫)), this was not as effective as when encapsulated cells were implanted (Figure 5A, (⟐) compared with (⚫)). Moreover, although ifosfamide and MESNA treatment was stopped around day 80, by day 98, there was already a statistically significant difference in the mean tumour volumes of the two treatment groups, and it appeared that by day 112, the tumours in mice given encapsulated cells plus ifosfamide and MESNA (Figure 5A (⟐)) had stopped growing, whereas tumours in mice given only ifosfamide and MESNA (Figure 5A (☐)) continued to grow, albeit initially at a lower growth rate than tumours in untreated mice until day 115. A similar analysis of the mean body weight of the treated animals revealed a statistically significant difference between the weight of animals in the presence (Figure 5B, Group 3 (△)) or absence (Figure 5B, Group 2 (☐)) of implanted CBT-4B10 cell-containing capsules by day 105. After day 115, the tumour growth rate in mice given only ifosfamide and MESNA seemed to increase.

These data suggest that encapsulated cells implanted in the vicinity of tumours can be used to locally activate multiple (15) doses of ifosfamide, apparently without damaging their own ability to convert the chemotherapeutic, for at least 80 days, and maybe even longer. The implication of this study is that future clinical trials in humans should be designed to include multiple rounds of ifosfamide treatment, rather than the two rounds that have been used to date. Multiple rounds of ifosfamide treatment in such studies may be expected to enhance median survival and may allow tumour shrinkage to such an extent as to allow surgical resection.

The use of metronomic, i.e., low-dose, long-term and frequently administered chemotherapy, has attracted renewed interest as a potential tool to fight certain types of cancers. Cyclophosphamide is one of the most widely investigated agents for such an approach, but ifosfamide has also been used in metonymic cancer treatments [38]. The main possible mechanisms of action identified in preclinical models, whatever the histology of the tumour, are the stimulation of the immune system and anti-angiogenic action [4]. However, the mouse models reported here are immunodeficient, so this cannot be the mechanism of action for the tumour reductions measured in the mice after repeated doses of ifosfamide. An anti-angiogenic action, however, can also be ruled out as a mechanism behind the observed tumour reductions since mice that received ifosfamide but no encapsulated cells showed only marginal and, in some cases, transient tumour reductions. As stressed by Muraro and colleagues, it is very unlikely that a single metronomic regimen could have universal efficacy for any given tumour type, and they support its use in combination with immune checkpoint inhibitors [39].

Future studies could focus on optimisation of the treatment, for example, by varying the dose of capsules given to each mouse, using different tumour cell lines (human and mouse) with slower- or faster-growing tumours, and trying newer agents from the oxazaphosphorine family besides ifosfamide.

## Figures and Tables

**Table 1:** IC_50_ of the five best performing clones compared to the standard CYP2B1-expressing cell line 22P1G, as evaluated via IFO-bioassays (XTT); XTT assay with 5 clones pre-selected for high enzymatic activity. A total of 5 × 10^3^ cells/well were seeded in quadruplicate into 96-well plates. Five days after the addition of 0, 0.005, 0.015, 0.05, 0.165 or 0.54 mM ifosfamide, the XTT-reagent was added and the metabolic activity of cells was measured. The Inhibitory Concentration (IC_50_) of the clones was evaluated using Excel Fit. The IC_50_ reflects how much Ifosfamide (in mM) is needed to inhibit the metabolic activity of the cells by half.

	CBT Clones	IC_50_ (mM IFO)
1	CBT-4B10	0.0117 ± 0.0005
2	CBT-1B6	0.0181 ± 0.0014
3	CBT-4F5	0.0363 ± 0.0095
4	CBT-4H9	0.0394 ± 0.0019
5	CBT-3D7	0.0579 ± 0.0263
	22P1G	0.0480 ± 0.0032

**Table 2:** Encapsulated CBT-4B10-mediated product conversion rate of ifosfamide metabolites (ng/mL/min) [4].

Metabolite	250 µM Ifosfamide (65.25 µg/mL)	3 mM Ifosfamide (783 µg/mL)
4-hydroxyfosfamide (+aldoifosfamide)	18.6 ± 1.5	104.6 ± 1.5
2-dechloroethylifosfamide	6.75 ± 1.2	32.2 ± 4.0
3-dechloroethylifosfamide	8.31 ± 2.2	39.1 ± 4.2
Free acrolein	23.64 ± 4.4	59.6 ± 4.0

## Data Availability

The data presented in this study are available on request from the corresponding author.
